# LES/CMC Simulations of Swirl-Stabilised Ethanol Spray Flames Approaching Blow-Off

**DOI:** 10.1007/s10494-016-9762-1

**Published:** 2016-11-09

**Authors:** Andrea Giusti, Maria Kotzagianni, Epaminondas Mastorakos

**Affiliations:** 1 Department of Engineering, University of Cambridge, Trumpington Street, Cambridge, CB2 1PZ UK; 2 Insitut für Energietechnik, ETH Zürich, ML L13, Sonneggstrasse 3, CH-8092 Zürich, Switzerland

**Keywords:** Large eddy simulation, Conditional moment closure, Turbulent spray flames, Local extinction, Lift-off

## Abstract

Large Eddy Simulations (LES) with the Conditional Moment Closure (CMC) combustion model of swirling ethanol spray flames have been performed in conditions close to blow-off for which a wide database of experimental measurements is available for both flame and spray characterization. The solution of CMC equations exploits a three-dimensional unstructured code with a first order closure for chemical source terms. It is shown that LES/CMC is able to properly capture the flame structure at different conditions and agrees reasonably well with the measurements both in terms of mean flame shape and dynamic behaviour of the flame evaluated in terms of local extinctions and statistics of the lift-off height. Experimental measurements of the overall (liquid plus gaseous) mixture fraction, performed using the Laser-Induced Breakdown Spectroscopy technique, are also included allowing further assessment and validation of the numerical method. The sensitivity of the simulation results to the various boundary conditions is discussed.

## Introduction

Spray flames approaching blow-off are characterized by strongly unsteady phenomena which can be considered quite challenging to be reproduced in numerical simulations. Flame-turbulence interactions and spray evolution should be properly taken into account in order to have a reliable evaluation of the unsteady behavior of the flame. Our simulation capability of ignition and extinction phenomena is not fully validated yet, not least because capturing the local extinction and its evolution into a global blow-off has not been extensively demonstrated with current generation turbulent combustion models. Local extinction of non-premixed flames in LES has been dealt with in advanced combustion models (such as CMC, Eulerian transported PDF, and flamelet-progress variable) and, in particular, the degree of local extinction in the Sandia piloted jet series seems reasonably well predicted (e.g. [10, 13, 14]). However, similar capability for spray combustion CFD has not been demonstrated yet, except for a LES/CMC attempt [27] that qualitatively showed some success in capturing the localised extinction seen in the n-heptane spray experiment of Cavaliere et al. [5]. In this work, further validation of LES/CMC is attempted by comparing the flame structure of an ethanol spray flame with a wide range of experimental measurements [31] concerning flame sheet location at different air flow conditions. Experiments also include Mie scattering and droplet size and velocity measurements, allowing hence a detailed study of the sensitivity of the simulations to the spray boundary conditions, an area introducing significant uncertainty in liquid-fuelled combustion CFD. In order to further assess the capability of the present numerical approach to predict the complex interactions between the evaporating spray and the reacting field, novel measurements of the overall (i.e. liquid plus vapour) mixture fraction, made possible by the Laser Induced Breakdown Spectroscopy (LIBS) technique, are also presented and compared with the numerical results.

The paper is organized as follows. First, the investigated burner is presented together with a description of the experimental method used for the LIBS measurements. The numerical approach and models used in the simulation are then introduced highlighting the main assumptions and features of the adopted approach. This is followed by the presentation and discussion of the results. Finally, conclusions and recommendations for future research are given.

## The Investigated Burner and Experimental Methods

**Fig. 1:**
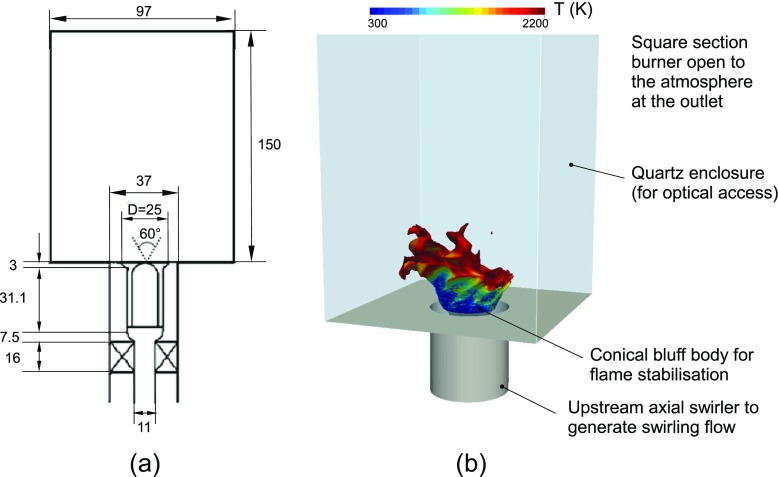
*Left:* schematic of the investigated burner (all the dimensions are in mm). *Right:* description of the main parts of the burner (an instantaneous isosurface of the stoichiometric mixture fraction coloured with temperature from LES is included)

The burner investigated in the present work (see Refs. [31, 32] for details) consists of a pressure atomizer (hollow-cone output profile and nominal spray angle of 60 ^∘^), fitted to a conical bluff-body holder, which injects the fuel into a square section enclosure open to the atmosphere at the outlet. A swirled air flow (swirl number SN=1.23 according to the definition of Ref. [2]), supplied through the annular duct surrounding the bluff body, allows the generation of a swirl-stabilized flame. The presence of a recirculation zone induced by the strongly swirling air (with the additional contribution due to the bluff-body geometry) allows us to mimic the basic features of gas turbine and industrial furnace flames making this test case relevant also for industrial applications. Figure 1 shows a schematic of the burner with a summary of the main parts.

Experimental measurements include high speed OH* chemiluminescence, OH-PLIF and Mie scattering diagnostics and PDA measurements for both flame and spray characterization. A detailed description of the experimental methods for such measurements can be found in Ref. [31]. In addition to that, new experiments based on the LIBS technique were performed, allowing us to characterize the overall mixture fraction of the reacting field. For the LIBS measurements, the focused (f=75mm) laser beam of a 6 ns Q-switched Nd:YAG laser (Continuum Surelite) operating at 1064 nm was used to induce the dielectric breakdown of the mixture. The employed energy was 140mJ, which was able to ensure the plasma formation everywhere in the combustor. The emitted radiation was collected using a plano-convex lens (f=50mm) and then focused into an optical fibre attached to a portable low cost spectrometer (Ocean Optics, USB2000), covering the spectral range 178-877 nm with 0.34 nm resolution. The acquisition of the emitted plasma radiation was performed 8 μs (time delay, *t*_*d*_) after the plasma creation and for 3 ms (time width, *t*_*w*_) so as to enhance the signal to noise ratio of the measurements and also avoid any contribution from the Bremsstrahlung radiation which is intense at the first stages of the plasma creation. In order to eliminate any contribution originating from the soot emission and the flame luminosity, the background emission from the environment was also subtracted from the measurements (i.e. the intensity of the spectral lines). For the creation of the calibration curve, LIBS measurements were performed within known uniform dispersions of ethanol droplets in air at various concentrations, while the ratios *H*_*α*_(646.3 nm)/O(777.3 nm) and *C*_2_(516.5 nm)/CN(388.3 nm), as proposed in Refs. [17, 33], were employed as indicators for the measurement of the local overall fuel-to-air ratio (i.e. without distinguishing whether the fuel is in liquid or vapour form since the large laser energy ensured complete evaporation of the droplets in the plasma volume). The ratio *H*_*α*_/O was used for overall mixture fraction *ξ*_*ov*_<0.1 whereas the ratio *C*_2_/CN was considered for *ξ*_*ov*_>0.1 (see Ref. [33] for details on the calibration procedure).

## Numerical Methods

Spray simulations performed in this work are based on a two-way coupled Eulerian-Lagrangian method for dilute sprays. The gas phase was solved using the Large Eddy Simulation (LES) approach whereas combustion was modelled by means of the Conditional Moment Closure (CMC) combustion model [16, 20, 21]. In the following, the main modelling assumptions adopted in this work are summarized with particular attention to the CMC model.

### CMC modelling

The CMC model is based on the solution of the conditionally filtered mass fraction of species and the conditionally filtered enthalpy or temperature. The CMC equation for a generic reacting scalar can be expressed as:

1 $$ \frac{\partial Q_{\alpha}}{\partial t} + \widetilde{u_{j}|\eta}\frac{\partial Q_{\alpha}}{\partial x_{j}}=e_{\alpha} + \widetilde{N|\eta}\frac{\partial^{2} Q_{\alpha}}{\partial \eta^{2}}+\widetilde{\omega_{\alpha} |\eta}+S(\widetilde{\Pi|\eta}) $$

where $Q_{\alpha }=\widetilde {Y_{\alpha } |\eta }$ is the filtered conditional mass fraction for the *α*-th species (*η* is the sample space variable in mixture fraction space). The unconditional value of a generic filtered quantity $\widetilde {f}$ can be computed from the respective conditional values $\widetilde {f|\eta }$ by means of a Filtered probability Density Function (FDF):

2 $$ \widetilde{f} = {{\int}_{0}^{1}}\widetilde{f|\eta} \widetilde{P}(\eta)d\eta $$

where $\widetilde {P}(\eta )$ is the FDF. In this work, the FDF was presumed to have a *β*-function shape computed from the resolved mixture fraction, $\widetilde {\xi }$, and its variance $\widetilde {\xi ^{\prime \prime 2}}$. Looking at the terms in Eq. 1, *e*_*α*_ represents the contribution from the sub-grid scales. This term was modelled using the typical gradient assumption, neglecting the contribution involving the sub-grid conditional joint fluctuations of the droplet evaporation rate and species [3, 27, 29]. For the conditional velocity, $\widetilde {u_{j} |\eta }=\widetilde {u_{j}}$ was assumed whereas $\widetilde {N|\eta }$ was closed with the Amplitude Mapping Closure (AMC) model [22]: $\widetilde {N|\eta }=N_{0}G(\eta )$, with *G*(*η*)= exp(−2[erf^−1^(2*η*−1)]^2^) and $N_{0}=\widetilde {N}/{{\int }_{0}^{1}} G(\eta )\widetilde {P}(\eta )d\eta $. The filtered scalar dissipation rate $\widetilde {N}$ is computed starting from the LES solution of the gas phase and consists of contributions from both resolved and sub-grid fields:

3 $$ \widetilde{N}=D\frac{\partial \widetilde{\xi}}{\partial x_{i}}\frac{\partial \widetilde{\xi}}{\partial x_{i}}+\frac{1}{2}C_{N}\frac{\nu_{t}}{{\Delta}^{2}} \widetilde{\xi^{\prime\prime 2}} $$

where $D=\mu /(\overline {\rho }Sc)$ is the molecular diffusivity (a Schmidt number equal to 0.7 was assumed), *ν*_*t*_ is the sub-grid scale kinematic viscosity and *C*_*N*_ is a model constant (see Ref. [26] for a discussion of all these models). The constant *C*_*N*_ was taken equal to 42.0. This value, obtained through matching computational and experimental results in Sandia D flame [10], gave good predictions of local extinction in both gaseous and spray flames [27, 34, 35] when used in conjunction with an algebraic model for the mixture fraction variance. Although we use here the same constants, the application to cases involving sprays needs further assessment, especially when the sub-grid mixture fraction variance is dominated by the effects of spray evaporation. The term $S(\widetilde {\Pi | \eta })$ in Eq. 1 represents conditional source terms related to spray evaporation which were modelled following the strategy discussed in [27]. Finally, first order closure was used for the chemical source term $\widetilde {\omega _{\alpha } |\eta }$.

### Chemistry modelling

**Fig. 2:**
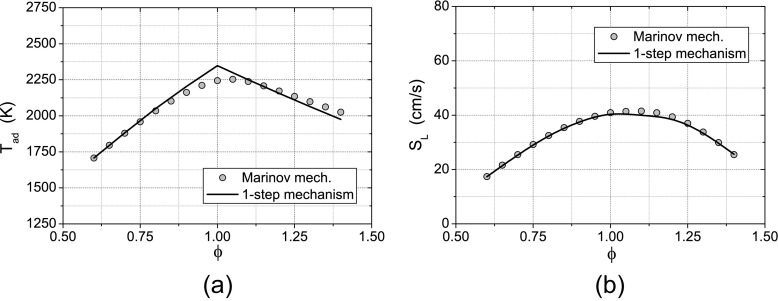
Comparison between the 1-step chemistry model and detailed chemical mechanism [18] in terms of adiabatic flame temperature obtained from chemical equilibrium computations and laminar flame speed in a freely propagating flame configuration (computations were performed with the software Cosilab ^*®*;^ [6])

In order to reduce the computational cost associated with the CMC model, a modified one-step chemistry model, developed following the method proposed by Fernández-Tarrazo et al. [8], was used. This method (the procedure, described for a generic hydrocarbon, was extended to alcohols) is based on the tuning of heat release rate and activation temperature of the one-step mechanism as a function of the local equivalence ratio in order to obtain approximately the correct laminar flame speed and adiabatic flame temperature across the whole flammable range. The calibration of the modified one-step chemistry model was performed by taking results from a detailed mechanism [18] as reference. In Fig. 2 the adiabatic flame temperature and the laminar flame speed of the resulting mechanism are compared with the values predicted by the detailed mechanism for several values of the equivalence ratio *ϕ* (please note that the activation temperature of the one-step mechanism was calibrated to interpolate the laminar flame speed of the detailed mechanism for both lean and rich equivalence ratios). The developed modified one-step mechanism is summarized in Table 1. This mechanism exhibits a critical scalar dissipation rate *N*_0,*cr*_, evaluated by solving the CMC equations without transport in physical space and spray source terms and with a prescribed *N*_0_ (the so called 0D-CMC solution which is representative of a transient flamelet with unity Lewis number and a given distribution of the scalar dissipation rate in the mixture fraction space), equal to 267 1/s, not too far from the one obtained with the detailed chemical mechanism (367 1/s). The smaller value of critical scalar dissipation rate could lead to an overestimation of the amount of local extinction, however it should be noted that similar differences can also be obtained by comparing different detailed mechanisms. In order to take into account all the features of the modified one-step chemistry model, the conditionally filtered energy equation in the CMC model was expressed in terms of conditional temperature. This allows the effects of the variation of the heat of reaction as a function of the local equivalence ratio to be directly included into the chemical source term appearing in the temperature equation.

**Table 1:** Modified one-step mechanism developed in this work

Reaction rate *k*, mol/(*m* ^3^s):		$k=\beta [C_{2}H_{5}OH][O_{2}]\exp (-T_{a}/T)$	
Activation temperature *T*_*a*_, K:			
	for *ϕ*<0.85	*T*_*a*_ = *T*_*a*0_[1.0+0.0432(0.85−*ϕ*)+0.517(0.85−*ϕ*)^2^]	
	for 0.85≤*ϕ*≤1.1	*T*_*a*_ = *T*_*a*0_	
	for *ϕ*>1.1	*T*_*a*_ = *T*_*a*0_[1.0−0.215(*ϕ*−1.1)+1.35(*ϕ*−1.1)^2^]	
Heat of reaction *Q*_*c*_, J/mol:			
	for *ϕ*≤1.0	*Q*_*c*_ = *Q*_0_	
	for 1.0<*ϕ*≤(*α*+1.0)/*α*	*Q*_*c*_ = *Q*_0_[1.0−*α*(*ϕ*−1.0)]	
	otherwise	*Q*_*c*_=0	
Other quantities and parameters:		*α*	0.325
		*β*, *m* ^3^/(mol s)	5.5×10^7^
		*Q*_0_, J/mol	1277.38×10^3^
		*T*_*a*0_, K	12000

### Flow field modelling

The LES equations with the low Mach number approximation were considered for the gas-phase. Since the CMC model is based on a mixture fraction approach, besides the equations for the conservation of mass and momentum, an additional equation for the transport of the filtered mixture fraction, $\widetilde {\xi }$, is solved [27]:

4 $$ \frac{\partial \overline{\rho} \widetilde{\xi}}{\partial t} + \frac{\partial \overline{\rho} \widetilde{u}_{j} \widetilde{\xi} }{\partial x_{j}} = \frac{\partial}{\partial x_{j}}\Big(\overline{\rho}D \frac{\partial \widetilde{\xi}}{\partial x_{j}}\Big)+\frac{\partial J_{SGS}}{\partial x_{j}}+\overline{\rho}\widetilde{\Pi} $$

where *D* is the molecular diffusivity and $\widetilde {\Pi }$ represents the source term due to droplet evaporation. A gradient model was used for the sub-grid term, $J_{SGS}= \overline {\rho } D_{t} \partial \widetilde {\xi }/\partial x_{j}$, with the turbulent diffusivity computed from the turbulent viscosity assuming a turbulent Schmidt number equal to 0.4 [15, 24, 26, 27]. The sub-grid scale (SGS) stress tensor was closed using the constant Smagorinsky model with *C*_*S*_=0.17.

Detailed attention should be devoted to the computation of the sub-grid scale mixture fraction variance, $\widetilde {\xi ^{\prime \prime 2}}$, since, as discussed in Section 3.1, this quantity is directly involved in the the computation of both the FDF and the sub-grid scale scalar dissipation rate. The variance of the mixture fraction field can be computed by means of either a transport equation or an algebraic closure [23, 26]. The modelling of the effects of spray evaporation on the sub-grid mixture fraction variance is still an open question. Due to lack of consensus on their modelling, the spray related terms are often neglected (e.g. [29, 30]), however there is evidence from Direct Numerical Simulation (DNS) studies that the effect of spray evaporation on the mixture fraction variance could not be negligible when the evaporation rate is not low and therefore the turbulent mixing is not the dominant process [25]. In the context of dilute sprays, Pera et al. [23] proposed an algebraic model, based on the equilibrium assumption, which also accounts for spray evaporation:

5 $$ \widetilde{\xi^{\prime\prime 2}} = C_{V}^{e**}\Bigg(C_{V}^{e*}{\Delta}^{2} \frac{\partial \widetilde{\xi}}{\partial x_{i}}\frac{\partial \widetilde{\xi}}{\partial x_{i}} + \frac{{\Delta}^{2}}{\nu_{t}} \widetilde{\xi ^{\prime\prime}{\Pi}^{\prime\prime}}\Bigg) $$

where $C_{V}^{e*}$ and $C_{V}^{e**}$ are two model parameters to be determined with a dual-step dynamic procedure and $\widetilde {\xi ^{\prime \prime }{\Pi }^{\prime \prime }}$ is the term representing the effects of spray evaporation (this term requires a proper closure). Assessment against DNS data [23] revealed that, although the model is able to reproduce quite well the trend of the sub-grid mixture fraction variance, a proper agreement with the reference DNS solution is quite troublesome, even in simple homogeneous flows. Further assessment and development is therefore necessary especially for applications involving complex flow structures. A formulation based on the algebraic closure of Eq. 5 with constant values for the model parameters, was recently used by Tyliszczak et al. [27]: $C_{V}^{e*}$ was assumed to be equal to 0.1 (following the suggestions of Branley and Jones [4] for gaseous flows) whereas $C_{V}^{e**}$ was arbitrarily set equal to 1.0 [28]. The contribution from the disperse phase, $\widetilde {\xi ^{\prime \prime } {\Pi }^{\prime \prime }}=\widetilde {\xi {\Pi }}-\widetilde {\xi }\widetilde {\Pi }$, was closed with a model similar to the one proposed by Demoulin and Borghi [7]. In regions located along the spray trajectory and characterized by the presence of a large amount of evaporating droplets, the term related to spray evaporation could give a great contribution to the overall value of the mixture fraction variance and the choice of model parameters could have a important effect on the solution. As previously stated, the modelling of the sub-grid mixture fraction variance in the presence of an evaporating spray is still an open issue and further developments together with a more rigorous validation of the modelling strategies (including the model used for the term $\widetilde {\xi ^{\prime \prime }{\Pi }^{\prime \prime }}$) appear necessary in order to have a more reliable formulation.

In this work, the formulation used by Tyliszczak et al. [27] was adopted (the use of constant values for the modelling parameters is also consistent with the use of a constant Smagorinsky model for the SGS stress tensor). It is important to point out that the use of a Lagrangian approach for particle tracking together with an algebraic closure for the sub-grid scale mixture fraction variance could lead to the presence of peaks in the variance field caused by the discrete distribution of particles, and therefore evaporation source terms, inside the domain. This could cause numerical instabilities when the unconditional density is given back to the LES code (see Section 3.4 for a description of the coupling strategy) making necessary the use of smoothing techniques for the spray term in the variance field. Such issues could be overcome using a transport equation for the sub-grid scale mixture fraction variance and this will be attempted in future work.

### Solution strategy and numerical models

The LES code PRECISE-UNS (see Ref. [1] for details) was used for the solution of the spray and gas-phase evolution whereas the CMC equations were solved using a superimposed in-house unstructured code [11, 34]. The coupling between the two solvers is achieved through density and temperature and is based on the strategy described in [11] with additional features due to the presence of the spray. The CMC solver receives flow field quantities (including spray source terms) from the LES code and, after the solution of the CMC equations, gives back to the LES solver the unconditional density and temperature fields (together with additional quantities required by the spray sub-models). In order to further reduce the computational cost associated with the CMC model, the CMC equations are solved in a mesh coarser than the one used for the LES code. Several strategies [26] can be used to transfer the quantities computed at the LES resolution to the CMC mesh. In particular, in the present work, both the conditional scalar dissipation rate and the spray source terms at the CMC resolution were obtained through the FDF-weighted integration over each CMC cell of the respective values computed at the LES resolution. Full operator splitting method was used for the solution of the CMC equations. The transport in the physical space was solved first, followed by the diffusion in mixture fraction space with spray source terms included. First-order upwind scheme was used for convective terms whereas diffusion terms were discretized with second-order schemes. A first-order scheme was used for time discretization. Finally, the contribution due to the chemical source term was included using the VODPK implicit solver. As far as the LES solver is concerned, second-order accurate schemes were used for spatial discretization whereas second-order implicit backward scheme was used for time derivatives.

### Computational domain and boundary conditions

The computational domain reproduces the experimental rig with the air inlet located 40 mm upstream of the bluff-body edge, immediately downstream of the axial swirler. The LES mesh used in this work consists of approximately 4 million hexahedral cells, with a minimum grid size of about Δ=0.2 mm in the vicinity of the bluff body, whereas the CMC mesh consists of about 80,000 cells, refined in the flame region in order to properly capture the dynamic behaviour of the flame. As far as the discretization of the mixture fraction space is concerned, 51 nodes clustered around the stoichiometric mixture fraction were used. A prescribed velocity profile (both axial and swirl components) and a constant temperature were imposed at the air inlet. Simulations presented in this paper were performed without imposing velocity fluctuations at the inlet boundary. This choice was motivated by the fact that no velocity measurements are available at the inlet section (inside the annular duct upstream of the bluff-body) and therefore the level of fluctuations would have been a quite arbitrary quantity. Furthermore, assessment of the effect of velocity fluctuations imposed at the inlet boundary, performed in preliminary computations, showed that the turbulent fluctuations at the inlet mainly affect the region very close to the bluff-body edge being the level of turbulence farther downstream dominated by the velocity fluctuations generated by the strong swirling flow. A constant pressure condition was used at the outlet whereas all the solid surfaces were modelled as adiabatic walls with no-slip condition for the velocity. Refinement of the mesh close to the walls was adopted allowing a proper resolution of the boundary layer. As regards the CMC equations, *η*=0 corresponds to air and *η*=1 to pure vaporised fuel; the inert mixing solution was prescribed at the air inlet and a zero-gradient condition was applied at walls and outlet boundary. A time step equal to 0.003 ms was used for both the LES and CMC solvers. The simulations were performed on 48 2.6 GHz processors with 4 GB of RAM per processor. The computation of 1 ms of physical time could be completed in less than 5 h.

### Spray injection modelling

**Fig. 3:**
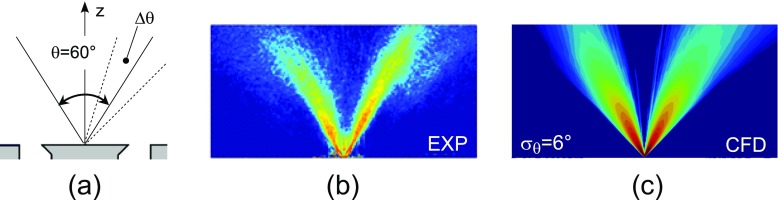
(**a**) Schematic of the injection strategy; (**b**) mean Mie scattering image in log-scale from experiments [31]; (**c**) mean Mie equivalent quantity predicted by numerical simulations in log-scale (Case E1S1, *U*_*b*_=17.1 m/s)

**Fig. 4:**
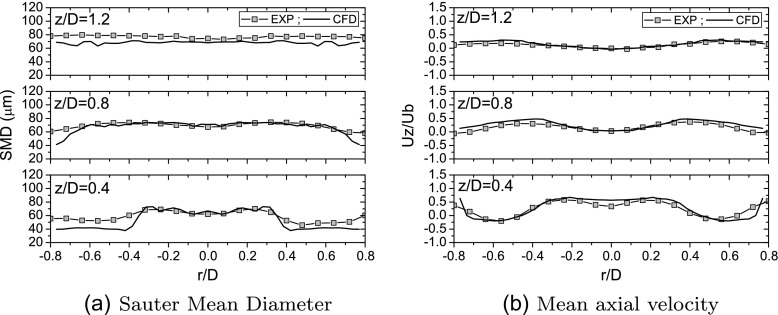
Comparison between numerical results and experimental measurements of mean axial velocity and Sauter Mean Diameter (SMD) [31] at different heights from the bluff-body surface - Case E1S1 (*U*_*b*_=17.1 m/s)

Experiments showed that in the case of the flame under investigation the injector does not behave as a perfect hollow cone and droplets with positive axial velocity (towards the outlet) are detected close to the injector axis (see Figs. 3 and 4). These droplets are likely to come directly from the injector as can also be deduced from Mie scattering images which show a high dispersion of droplets around the nominal cone angle (see Fig. 3b). In order to reproduce this behaviour, the spray injection was modelled with a stochastic model. The hollow cone was reproduced by assigning a mean injection angle and a random component added to it, sampled from a truncated normal distribution. A sensitivity analysis to the dispersion around the mean angle was performed (in the following, the symbol *σ*_*𝜃*_ will be used to indicate the standard deviation of the spray half cone angle). As far as the droplet diameter is concerned, analysis of the PDA measurements along the spray path at *z*/*D*=0.4, suggested that the droplet size volume distribution can be reasonably approximated with a Rosin-Rammler function with Sauter Mean Diameter (SMD) and dispersion parameter *q* in the range 60−70 μ*m* and 3.0−4.0, respectively. In all the simulations presented in this work, droplet diameters were computed from a Rosin-Rammler distribution with SMD equal to 60 μm and *q*=3.0. The injection velocity magnitude, taken equal for all the droplets, was calibrated in order to have a reasonable agreement with the experiments at the first measurement location (i.e. *z*/*D*=0.4). Spray evaporation was accounted for by means of a uniform temperature model with equilibrium assumption for the computation of the fuel vapour mass fraction at the droplet surface [19]. No secondary breakup model was used due to the low value of droplet Weber number [31]. Air and fuel mass flow rates and temperatures were set up according to experimental conditions [31].

## Results and Discussion

In the following, the results obtained with the LES/CMC method will be compared with experimental measurements [31]. Two cases, characterized by the same fuel mass flow rate (0.27 g/s) but different air-flow bulk velocity (*U*_*b*_), will be considered. Both these cases represent flames close to blow-off where the experiments showed a large amount of local extinction. First, the capability of the present approach to capture the spray evolution will be analysed through comparisons with Mie scattering and PDA measurements for one of the investigated cases. Then, comparisons in terms of both instantaneous and mean flame shape will be discussed followed by the mean overall mixture fraction. Finally comparisons between numerical and experimental statistics of the lift-off height from the outer anchoring part will be shown, to provide an assessment of the accuracy of the model to capture the degree of local extinction there.

### Spray comparisons

In Fig. 3 the experimental mean Mie scattering image is compared with the time average of a numerical equivalent quantity proportional to the sum of the surface of droplets crossing each cell. Considering a generic i-th cell, the local value of this quantity at a given time step was computed as:

6 $$ \text{Mie}^{(eq)}_{i}(t)\propto \frac{{{\sum}_{k=1}^{N}} n_{k} {d^{2}_{k}}}{V_{i}} $$

where *N* is the number of parcels crossing the cell, *n*_*k*_ is the number of real droplets in the *k*-th parcel, *d*_*k*_ is the diameter of the droplets in each parcel and *V*_*i*_ is the volume of the cell. It is evident that both the location and the spreading of the spray are quite well reproduced. Figure 4 compares the LES predictions of Sauter Mean Diameter and droplet mean axial velocity with experimental PDA measurements [31] for one of the investigated cases. Numerical results are in good agreement with the experiments demonstrating the capability of spray and injection modelling to correctly reproduce the main features of the spray in terms of mean velocity and diameter at different distances from the bluff-body surface.

### Instantaneous results

**Fig. 5:**
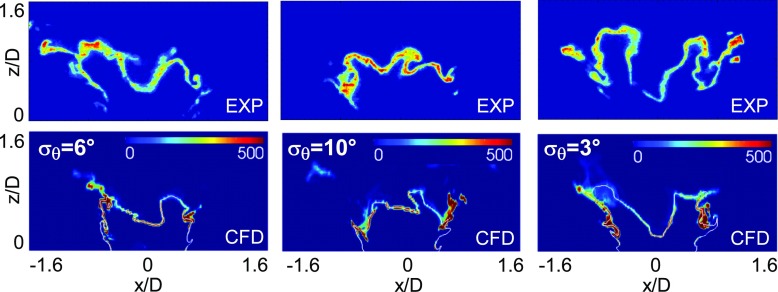
Comparison between instantaneous experimental [31] OH-PLIF images (*top row*) and instantaneous heat release rate (MW/ *m* ^3^) from CFD (*bottom row*, snapshots from simulations with different values of *σ*_*𝜃*_ are shown) - Case E1S1, *U*_*b*_=17.1 m/s

In Fig. 5 the instantaneous heat release rate from the simulations is compared with instantaneous OH-PLIF images from the experiment [31] for a case characterized by an air-flow bulk velocity equal to 79.2 % of the blow-off velocity. The LES results show a flame shape very similar to the one observed in the experiment. The stoichiometric mixture fraction iso-line is attached to the bluff-body edge (see the white line in Fig. 5), however the flame appears lifted-off as revealed by the negligible heat release rate (and also low temperature, see for example the stoichiometric mixture fraction isosurface included in Fig. 1) in this region. Therefore, in this case, the lift-off seems to be due to the incoming air flow which causes localized extinction along the flame brush. Both the transport in physical space and diffusion in mixture fraction space terms in the CMC equation could give important contributions to the local extinction around the bluff-body edge. A sensitivity analysis of the flame shape to the spreading angle of the spray cone was also performed (the snapshots reported in Fig. 5 come from simulations performed with different values of standard deviation, *σ*_*𝜃*_, of the half cone angle as indicated in the caption) showing that an increase of the dispersion around the mean angle seems to promote the destruction of the ‘V’ shape and the formation of a rounded flame. This finding could give a possible explanation to the rupture of the ‘V’ shape also observed in the experiments [31] which could be related to an increase of the number of droplets injected in the center of the cone.

**Fig. 6:**
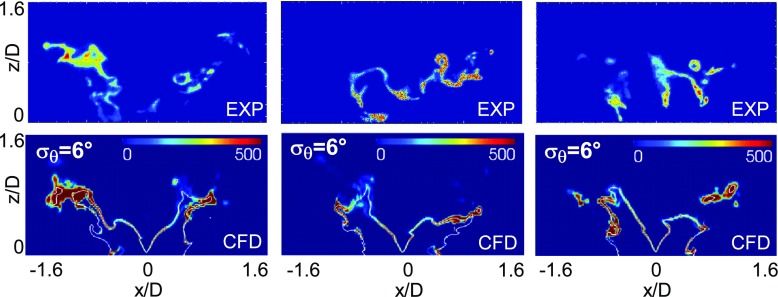
Comparison between instantaneous experimental [31] OH-PLIF images (*top row*) and instantaneous heat release rate (MW/ *m* ^3^) from CFD (*bottom row*) - Case E1S2, *U*_*b*_=20.0 m/s

Figure 6 shows comparisons between experimental and numerical results in a condition much closer to blow-off than the case in Fig. 5 (the air-flow bulk velocity is now equal to 92.6 % of the blow-off velocity). The experiment shows that moving closer to blow-off the flame becomes more fragmented with formation of several small pockets of OH. The inner reaction zone is less anchored to the spray and sometimes moves towards the outer flame brush. Numerical simulations are able to predict the more corrugated and fragmented structure of the flame as well as the formation of several pockets of reacting regions. Some discrepancies arise in the inner reaction zone where the degree of local extinction seems not correctly predicted. It is important to note that the inner flame brush lies in a region very close to the injection location and the spray path where the flame evolution could be strongly influenced by the spray evaporation making the numerical prediction quite challenging since it involves many modelling aspects that still need to be developed. As already discussed, the closure of spray terms in the sub-grid mixture fraction variance needs further development and validation. In addition to that, the presence of evaporating droplets could also have a direct impact on the shape of both the FDF and the conditional scalar dissipation rate and the use of the models employed in the current formulation (that is presumed *β*-function shape for the FDF and AMC model for the conditional scalar dissipation rate) may be questionable when the evaporation rate is not negligible. Furthermore, it should be noted that the flame is anchored very close to the injection location where the spray is still dense. The present Lagrangian formulation is based on a dilute spray assumption and therefore the models used to predict the spray evolution are not fully consistent in this region.

### Mean fields

**Fig. 7:**
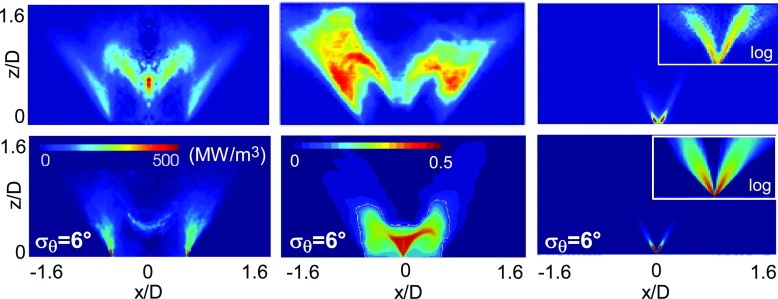
*Top row:* Experimental data [31] for the inverse Abel-transformed time-averaged OH* (*left*), mean OH-PLIF (*middle*), and mean Mie scattering image (*right*, image with intensity in log-scale is enclosed). *Bottom row:* LES mean heat release rate (*left*), mean gaseous mixture fraction (*middle*, the white line is the stoichiometric mixture fraction) and mean “MIE equivalent” (see text) quantity (*right*, image with intensity in log-scale is enclosed). Case E1S1, *U*_*b*_=17.1 m/s

**Fig. 8:**
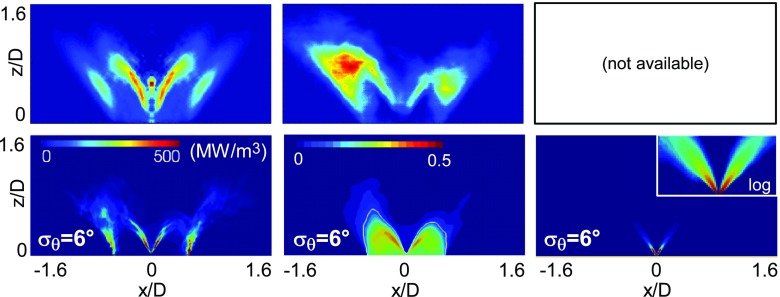
*Top row:* Experimental data [31] for the inverse Abel-transformed time-averaged OH* (*left*), mean OH-PLIF (*middle*), and mean Mie scattering image (*right*, image with intensity in log-scale is enclosed). *Bottom row:* LES mean heat release rate (*left*), mean gaseous mixture fraction (*middle*, the white line is the stoichiometric mixture fraction) and mean “MIE equivalent” (see text) quantity (*right*, image with intensity in log-scale is enclosed). Case E1S2, *U*_*b*_=20.0 m/s

Figures 7 and 8 show comparisons between numerical simulations and experiments [31] in terms of mean values for the two conditions analysed in the previous section. The inverse Abel-transformed time-averaged OH* from the experiments [31] is compared with the numerical mean heat release rate. Mean OH-PLIF images from experiments and mean mixture fraction fields from numerical simulations are also included together with Mie scattering images. Increasing the air-flow bulk velocity, and therefore moving closer to the blow-off, the flame becomes shorter with both the inner and outer branches lying closer to the bluff body. The numerical results reproduce this trend. Some discrepancies arise in the inner flame region where the flame front predicted by the numerical simulation appears less thick compared to the experiment and the ‘V’ shape is not perfectly reproduced. As already pointed out, the inner flame region is expected to be characterized by a strong coupling with the evaporating spray and some developments are still needed to improve the prediction capability in this region. However, the results can be considered satisfactory and demonstrate the overall capability of the current model to predict the mean behaviour of the flame approaching blow-off.

**Fig. 9:**
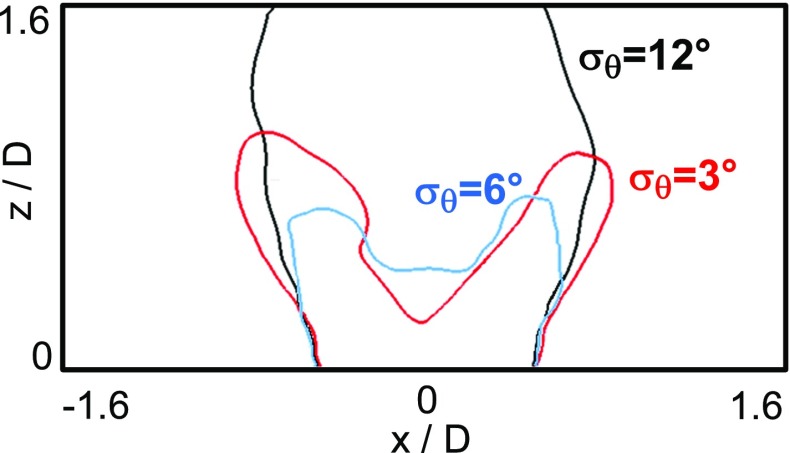
Mean stoichiometric mixture fraction iso-line for different values of *σ*_*𝜃*_ (Case E1S1, *U*_*b*_=17.1 m/s)

The effect of the spreading of the spray cone angle, previously discussed in terms of instantaneous fields (see Fig. 5), is further demonstrated in Fig. 9 where the mean stoichiometric mixture fraction iso-line predicted with different values of *σ*_*𝜃*_ is shown (case E1S1). The increase of the spreading angle increases the amount of fuel released in the region inside the spray cone leading to the formation of a rounded flame, which in the case of high values of *σ*_*𝜃*_ appears quite elongated along the injector axis. This further highlights the role exerted by the dispersion of droplets around the mean spray angle in determining the shape of this flame.

### Overall mixture fraction

In order to further assess the capabilities of the numerical simulation to reliably reproduce the main interactions between the evaporating spray and the reacting field, the overall mixture fraction was measured using the LIBS technique and compared to the numerical simulations. As already pointed out, the mixture fraction measured through the LIBS technique also includes the liquid fuel in the LIBS sample. In the numerical simulations, the overall mixture fraction, $\widetilde {\xi }_{ov}$, was determined starting from the resolved mixture fraction field and the total mass of droplets contained in each computational cell:

7 $$ \widetilde{\xi}_{ov}=\frac{\overline{\rho} \widetilde{\xi} + m_{d}^{*}}{\overline{\rho} + m_{d}^{*}} $$

where $m_{d}^{*}$ is the droplet mass density computed as the ratio between the total mass of droplets in a given cell and the volume of the cell.

**Fig. 10:**
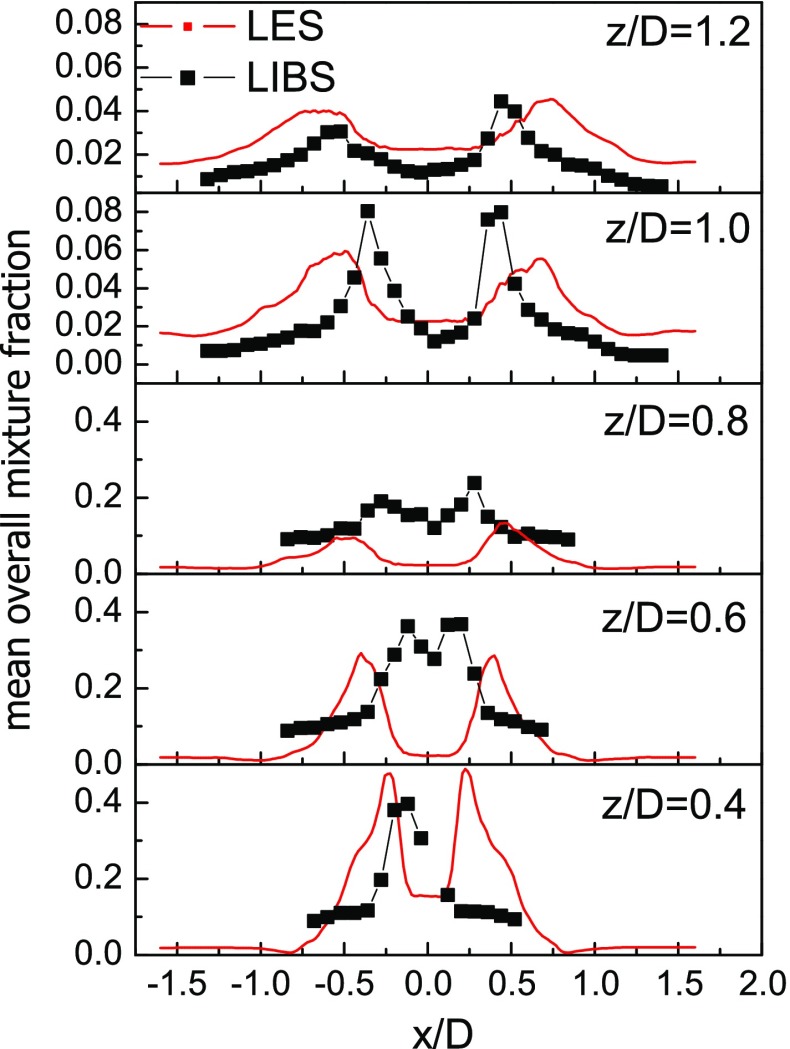
Comparison between numerical (*σ*_*𝜃*_=6^∘^) and experimental mean overall mixture fraction (Case E1S1, *U*_*b*_=17.1 m/s)

In Fig. 10, the overall mixture fraction radial profiles from LES are compared with the LIBS data (case E1S1). At the locations close to the injector, the overall mixture fraction is characterized by two different peaks. As the distance from the bluff-body increases, initially the two peaks become more distinguishable, but at even greater distances it is observed that the overall mixture fraction suggests a more uniform profile. This result is consistent with the flame shape observed in the OH-PLIF measurements with the peaks located in the middle between the inner and outer flame brushes. From the comparison of LIBS results to the images of Figs. 3 and 5, it is clear that both the location of the droplets (Mie image) and the reaction zone (OH images) can be well correlated to the intensity of the *C*_2_/CN and H _*α*_/O ratios respectively. On the basis of these ratios, high values of the overall mixture fraction were found close to the spray path whereas in the vicinity of the reaction zone the measurements revealed a composition close to stoichiometry, as expected.

**Fig. 11:**
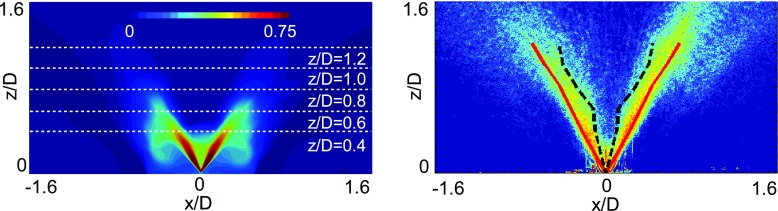
*Left:* LES predictions (*σ*_*𝜃*_=6^∘^) of the mean overall mixture fraction. *Right:* experimental Mie scattering image [31] in log-scale with location of the peaks of the overall mixture fraction superimposed; *black dashed line:* LIBS results; *red line:* LES results (Case E1S1, *U*_*b*_=17.1 m/s)

The LES results are in relatively good agreement with the experiment, with both the maximum value of the overall mixture fraction and its location being reasonably well predicted. Slightly larger deviations in both the peak location and the value of the overall mixture fraction inside the spray cone can be observed at intermediate distances from the injector. Several factors can contribute to these discrepancies, ranging from spray and chemistry modelling for the LES results to the inherent technical difficulties during the LIBS experiment (especially concerning the spatial resolution and the possibility that not all droplets evaporate at the LIBS plasma). By comparing the mean gaseous mixture fraction predicted by the LES (see Fig. 7) with the overall mixture fraction of Fig. 11, it is evident that the peaks in the region close to the injector are strongly affected by the presence of liquid droplets. This is consistent with the Mie scattering measurements which show the maximum intensity in the same region, as demonstrated in Fig. 11 where the location of the peaks of the overall mixture fraction from both LES and LIBS measurements are superimposed to the experimental Mie scattering image [31]. From Fig. 11 it is also possible to note that LIBS results tend to locate the peaks of the overall mixture fraction in the middle of the spray cone whereas the LES results, obtained by imposing a mean injection angle equal to the nominal spray cone angle of the injector, seem to slightly overestimate the opening of the spray. This explains the deviation between the LIBS and the LES in the prediction of the location of the peaks of the overall mixture fraction. Furthermore, from the comparisons shown in Fig. 7, it should be noted that the mean flame shape predicted by the LES appears slightly shorter compared to the experiment. This is consistent with the lower values of the overall mixture fraction predicted by the numerical simulations inside the spray cone at the intermediate sampling heights. As already pointed out, this region is highly influenced by the interaction between the evaporating spray and the reacting field and further developments are needed to improve the prediction capability in such region.

### Lift-off height

A very interesting metric for the evaluation of the capability of the present numerical approach to capture the dynamic behaviour of the flame is the statistical distribution of the lift-off height of the outer flame brush. As shown in Figs. 5 and 6, the experimental OH-PLIF images show an intermittent lift-off and lift-off height fluctuations, with the lift-off defined as the distance of the OH region from the corner of the bluff-body. Capturing the statistics of flame lift-off is considered a very challenging target for combustion CFD. Some success of the LES/CMC approach in capturing the probability density function of the lift-off height has already been shown in the case of swirling non-premixed gaseous flames [34]. Here the capability of the CMC model to capture the lift-off statistics is further evaluated in the context of spray flames.

**Fig. 12:**
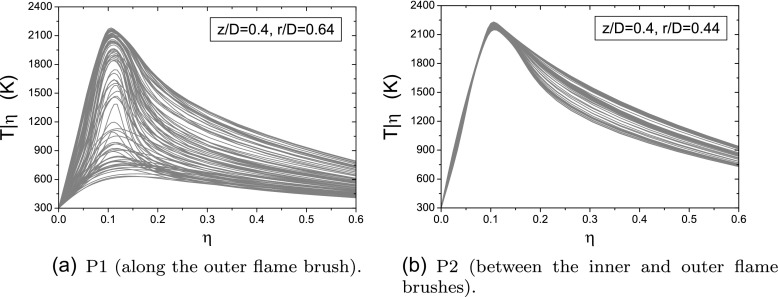
Time evolution (total time equal to 4.5 ms) of the conditionally filtered temperature at two different locations (Case E1S1, *U*_*b*_=17.1 m/s)

**Fig. 13:**
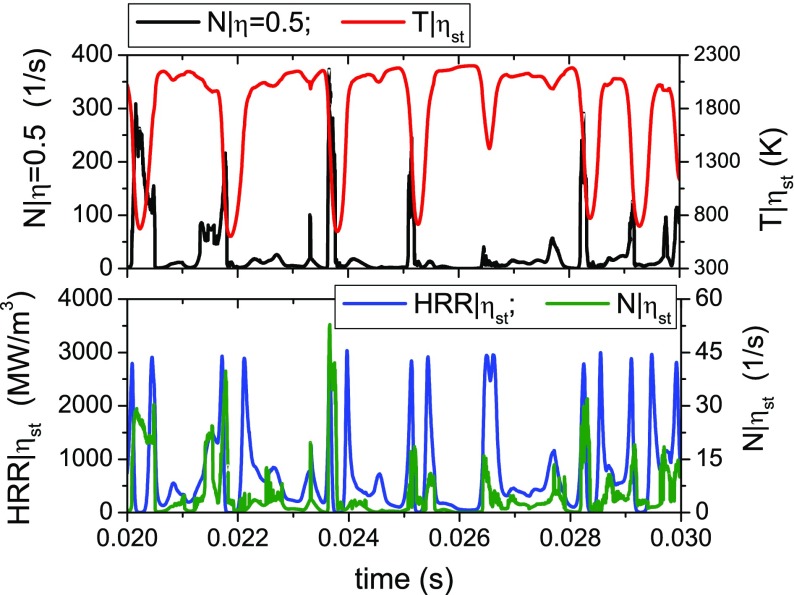
Time evolution of conditionally filtered temperature and heat release rate at the stoichiometric mixture fraction and conditionally filtered scalar dissipation rate at selected values of *η* for a CMC cell located at *z*/*D*=0.4, *r*/*D*=0.64 (Case E1S1, *U*_*b*_=17.1 m/s)

As already pointed out, in conditions close to blow-off the flame exhibits a high degree of local extinction so that the heat release rate close to the bluff-body edge could be negligible and the flame appears lifted-off. Figure 12 shows the time evolution of the conditionally filtered temperature in mixture fraction space at two different locations (CMC cells). The first one (P1) is representative of a location along the outer flame brush whereas the other one (P2) is located in the middle between the inner and the outer flame fronts (case E1S1). It is possible to see that along the outer flame front the conditional quantities evolve in time showing instantaneous extinctions and re-ignitions. This determines a variation in time of the lift-off height, consistent with the experiment where the outer flame brush is very variable, exhibiting subsequent detachments and reattachments to the bluff-body edge. On the contrary, points located far from the flame front and the spray injection location are characterized by small variations of the conditional quantities and the flame structure is close to a fully burning solution for all times. The local extinction and re-ignition behaviour along the outer flame brush is further analysed in Fig. 13 where the time evolution of specific conditional quantities is reported (case E1S1). Local extinction events are detected in the time intervals characterized by a low value of conditional temperature and negligible conditional heat release rate at the stoichiometric mixture fraction. It is interesting to note that local extinctions usually correspond to peaks of scalar dissipation rate. However, the value of the scalar dissipation rate is not necessarily higher than the critical value found in the stand-alone 0D-CMC computation (see Section 3.2). This suggests that local extinctions, although driven by the scalar dissipation rate, are also affected by the transport in physical space. Peaks of heat release rate appear during the ignition and extinction transients whereas the solution characterised by low heat release rate and high temperature identifies a fully-burning local flame structure at low scalar dissipation rate.

**Fig. 14:**
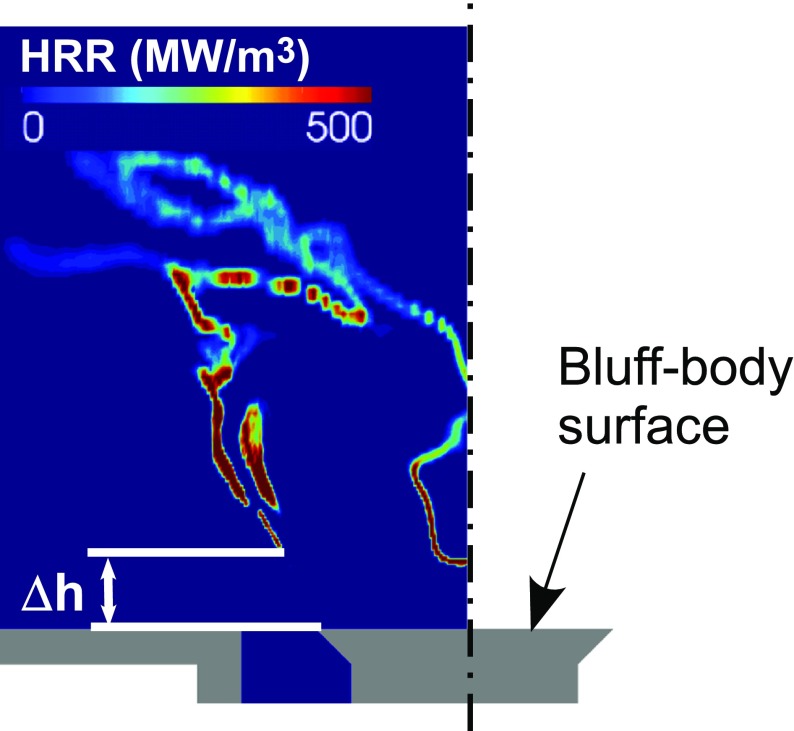
Definition of the lift-off height

**Fig. 15:**
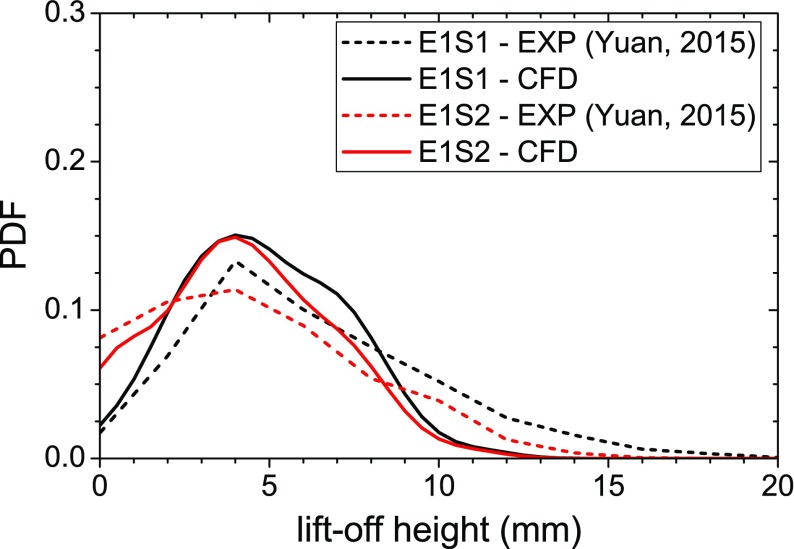
Statistics of the lift-off height: comparison between numerical results and experimental data [31]

A quantitative evaluation of the degree of local extinction along the outer flame brush can be performed by computing the probability density function of the lift-off height. As schematically shown in Fig. 14, the lift-off height was evaluated as the axial distance from the bluff-body surface at which non-negligible values of heat release rate (HRR) first appear along the outer flame brush in a stream-wise cross section. The analysis was performed considering data collected over a period of 12 ms with a sampling frequency of approximately 5 kHz. The procedure used to compute the lift-off height is equivalent to the one used in the experiments (see Ref. [31] for details) where the OH signal coming from OH-PLIF measurements was used as an indicator of the flame location in place of the HRR. Figure 15 shows comparisons between the numerical and experimental statistics of the lift-off height at the two conditions investigated in this work. Increasing the air-flow bulk velocity, the experiments show that (i) the mean lift-off height becomes shorter and (ii) the probability of reattachment of the flame front to the bluff body (i.e. lift-off height equal to zero) increases. The current LES/CMC method captured both these features with a good agreement between numerical and experimental results. Looking in detail at the lift-off height PDF in Fig. 15, the numerical simulation seems not able to predict the occasional very high values observed in experiments. Several factors can determine this behaviour, in particular the simple description of the chemistry based on the one-step mechanism and the absence of turbulent fluctuations at the inlet boundary which can promote the local extinction of the flame. However, the most probable value predicted by the numerical simulations is in good agreement with the experiment as well as the trend observed with the increase of the air-flow bulk velocity with an increase of the probability of flame reattachment. This suggests that the LES/CMC approach is very promising for capturing the behaviour of spray flames close to global extinction.

In order to further assess the capability of the LES/CMC approach to capture local extinctions in spray flames, detailed chemistry calculations of the same flame have been recently performed [12], also including a transport equation for the sub-grid scale mixture fraction variance, allowing a better description of the effect of spray evaporation on the sub-grid mixing field. The computation with the detailed chemical mechanism, performed with a CMC mesh of about 45,000 cells and a slightly different numerical setup, required almost 36 h to get 1 ms of physical time by using 128 2.6 GHz processors with 4GB of RAM per processor and the results showed a slightly improved lift-off height prediction. Although, the use of a detailed chemical mechanism allows a more accurate description of the turbulence-chemistry interaction, this shows that properly developed low-order chemistry models can still provide useful insights into complicated flame patterns [9]. Furthermore, the resulting quite small computational cost compared to detailed chemical mechanisms suggests the use of such models at least for preliminary computations, when the focus is on the main characteristics of the flame and details regarding the flame structure are not required.

## Conclusions

A swirling ethanol spray flame in conditions close to blow-off has been investigated using the LES/CMC approach with the main aim of further validating the capability of such method to capture local extinctions. Numerical results agree reasonably well with the experiments in terms of both instantaneous and mean flame shape at different air-flow bulk velocities. Increasing the air velocity, and therefore moving towards conditions close to blow-off, the flame becomes more fragmented and shorter, a behaviour well reproduced by the current LES/CMC method. The degree of local extinction of the outer flame brush appears reasonably well captured as demonstrated by comparisons with the lift-off height statistics obtained by the experiment. The prediction of flame detachment and reattachment to the bluff-body edge is in good agreement with experiments as well as the overall trend observed with the increase of the air-flow bulk velocity. Capturing the statistics of flame lift-off is considered a very challenging target for combustion CFD and it is very promising that the present method is reasonably successful in this respect.

The flame shape of the inner flame brush appears quite sensitive to spray boundary conditions as revealed by the sensitivity analysis to the spreading angle of the spray cone and comparisons with LIBS results. Furthermore, regions located along the spray path and characterized by a strong evaporation are expected to be very sensitive to the numerical models involving spray related terms. It should be noted that the models used in this study for the closure of the CMC equations are relatively simple. Future work should focus on the improvement of CMC sub-models involving spray evaporation for which further validation is still needed. In particular, the modelling of the sub-grid mixture fraction variance as well as the FDF and scalar dissipation rate in regions influenced by evaporation need further assessment and development.
